# Melatonin Suppresses Toll Like Receptor 4-Dependent Caspase-3 Signaling Activation Coupled with Reduced Production of Proinflammatory Mediators in Hypoxic Microglia

**DOI:** 10.1371/journal.pone.0166010

**Published:** 2016-11-03

**Authors:** Linli Yao, Pengfei Lu, Eng-Ang Ling

**Affiliations:** 1 School of Life Science and Technology, ShanghaiTech University, Shanghai, China; 2 Shanghai Institutes for Biological Sciences, Chinese Academy of Sciences, Shanghai, China; 3 Department of Anatomy, Yong Loo Lin School of Medicine, National University of Singapore, Singapore, Singapore; Universidade de Sao Paulo, BRAZIL

## Abstract

Microglia activation and associated inflammatory response play pivotal roles in the pathogenesis of different neurodegenerative diseases including neonatal hypoxic brain injury. Here we show that caspase3 expression was upregulated in activated microglia after hypoxic exposure, and remarkably, the cell viability remained unaffected alluding to the possibility of a non-apoptotic role of caspase3 in activated microglia. Chemical inhibition of caspase3 suppressed microglia activation as evident by an obvious reduction in expression of proinflammatory mediators and NF-κB signaling activation. Hypoxia induced caspase3 activation was TLR4 dependent as supported by the fact that caspase3 activation was hindered in cells with TLR4 knockdown. Interestingly, melatonin treatment significantly suppressed caspase3 activation. More importantly, melatonin also inhibited the increase in TLR4 protein and mRNA expression in hypoxic microglia. Inhibition of TLR4 expression by melatonin was also found in microglia of postnatal rats subjected to hypoxic exposure. Taken together, it is concluded that melatonin could inhibit TLR4 expression in hypoxic microglia followed by suppression of caspase3 activation leading to decrease in production of proinflammatory mediators.

## Introduction

The developing brain is highly vulnerable to oxygen deprivation or hypoxia [1, 2]. Neuroinflammation, that includes microglia over-activation, is considered as an important factor leading to the developmental deficiency in hypoxia [3]. It is well documented that activated microglia show increased production of free radicals, nitric oxide, glutamate and inflammatory cytokines. Overproduction of the neurotoxic and pro-inflammatory mediators can greatly amplify the brain injury in the hypoxic neonatal rats [1, 3–7]. Hence, controlling microglial activation is believed to be an important therapeutic strategy in suppression of neuroinflammation for amelioration of hypoxia induced brain damage. Therefore, clarifying the molecular mechanism of hypoxia induced microglia activation would be the first step towards controlling it. We have reported the involvement of Toll like receptor 4 (TLR4) in hypoxic microglia activation both *in vitro* and in hypoxic neonatal rats [8]. In this connection, a novel role of TLR4-dependent caspase-3 activation has recently been reported in activated microglia after stimulation with lipopolysaccharide (LPS) [9, 10]. It was reported that TLR4-dependent caspase3 activation could promote microglia activation in the absence of cell death. Remarkably, inhibition of caspase activation has been reported to protect against neuronal loss in hypoxic ischemia/stroke, suggesting an added role of caspase3 activation in hypoxic diseases [11]. However, it has remained to be ascertained whether caspase3 activation would contribute to microglia activation in hypoxia.

Melatonin (N-acetyl-5-methoxytryptamine), the major secretory product of the pineal gland, plays a fundamental role in the neuroimmuno-endocrine system. As a potent antioxidant, melatonin and its metabolites directly scavenge a variety of free radicals [12, 13]. There is ample evidence suggesting that melatonin possesses both antioxidative and anti-inflammatory effects [14–16]. Indeed, melatonin has been shown to inhibit microglia activation, and reduce pro-inflammatory cytokine levels in many experimental models including hypoxic brain injury in neonatal rats [17–19]. In hypoxic neonatal rats treated with melatonin, microglial cells showed a significant reduction in production of inflammatory cytokines when compared with corresponding cells in untreated hypoxic rats [19]. However, the mechanism through which melatonin acts on microglia activation is dubious. Studies have found that melatonin can inhibit TLR4 protein expression induced by ischemia/reperfusion in liver [20]. It was thus surmised that melatonin might exert its anti-inflammatory effect via suppression of TLR4 expression in activated microglia.

In light of the above, we first studied the activation of caspase3 in activated microglia after hypoxic exposure. The role of caspase3 in microglia activation was then investigated through blocking its activation by its specific inhibitor Z-DEVD-FMK followed by examining the expression of the major proinflammatory mediators and their related NF-κB p65 subunit activation. The function of TLR4 in caspase3 activation was then clarified by TLR4 knockdown. Furthermore, effects of melatonin supplement on caspase3 activation were evaluated in hypoxic microglia. TLR4 expression change and the underlying mechanism were investigated in melatonin treatment microglia *in vitro*. Additionally, the effect of melatonin on TLR4 expression was extended to developing rats subjected to hypoxic exposure.

## Materials and Methods

### Postnatal hypoxia and melatonin treatment

One day old Wistar rats were exposed to hypoxia following the protocols as reported previously [8]. Briefly, rats were placed in a chamber (Model MCO 18 M; Sanyo Biomedical Electrical Co, Tokyo, Japan) filled with a gas mixture of 5% O_2_ and 95% N_2_ for 2 h. They were then allowed to recover under normoxic conditions for 24 h before sacrifice. Age-matched control animals were kept outside the chamber. To assess the effect of melatonin in neonatal brain following hypoxic injury, rats were given intraperitoneal injections of melatonin (Sigma, MO, USA; catalogue number: M5250) dissolved in normal saline (10 mg/kg body weight) [19]. Each rat received the first injection of melatonin immediately before exposure to hypoxia, the second injection was immediately administered after it and the third injection 1 h after leaving the chamber. The experiments were repeated for three times. The project was approved by the Institutional Animal Care and Use Committee, National University of Singapore (IACUC number 095/08 (A2)11). All efforts were made to reduce the number of rats used and their suffering.

### BV-2 microglia subjected to hypoxia and melatonin treatment

BV-2 cells were used for *in vitro* study. The cells were cultured at 37°C in growth medium containing DMEM supplemented with 2% fetal bovine serum (FBS) (Invitrogen, Carlsbad, CA, USA) in a humidified incubator containing 5% CO_2_, and 95% air. The culture medium was changed to fresh medium for routine culture before the cells were exposed to hypoxia by placing them in a chamber filled with a gas mixture of 3% O_2_ + 5% CO_2_ + 92% N_2_ for 2, 4, 6, 8, 12 and 24 h. Melatonin (100 nmol) was added to the fresh medium 1h before hypoxia. The experiments have been repeated at least in triplicate.

### Immunofluorescence staining

TLR4 and caspase3 immunoexpresion was determined at 24 h after hypoxia. The rats were anesthetized with 6% sodium pentobarbital and perfused transcardially with 2% paraformaldehyde. After that, the brains were removed and post-fixed in 2% paraformaldehyde for 4 h and then cryoprotected in 30% sucrose overnight. Frozen sections at 40 μm were cut coronally with a cryostat (Model CM 3050; Leica Instruments GmbH, NUBLOCH, Germany) and mounted onto gelatin-coated slides. The sections were incubated with anti-rabbit TLR4 polyclonal antibody (1:100; Santa Cruz Biotechnology, catalogue number sc-10741) overnight followed with incubation with Cy3-conjugated secondary antibody 1 h the next day. The sections were again incubated with the FITC-conjugated lectin from tomato (Lycopersicon esculentum, 1:100, Sigma, MO, USA; Cat. No. L-0401) (1:100) and mounted using a fluorescent mounting medium with DAPI (Sigma, MO, USA, Cat. No. F6057). The images were captured under a confocal microscope (FV1000; Olympus, Tokyo, Japan). The optical density of TLR4 was analyzed with Image J software (National Institutes of Health, NIH, USA). Caspase 3 immunofluorescence was detected on BV-2 cells after hypoxia challenge for 8 h. BV-2 cells were fixed with 4% paraformaldehyde for 20 min and processed as described above for localization of Caspase 3 (1:100, Cell signaling, catalogue number 9661). All the experiments have been repeated for three times.

### Silencing of TLR4 with small interfering RNA (siRNA)

TLR4 expression was silenced using TLR4 small interfering RNA (siRNA) (Ambion, Foster City, CA, USA, catalogue number s75207) according to the manufacturer’s instructions. Non-treated BV-2 cells and BV-2 cells transfected with nonspecific scramble siRNA that does not target any mouse genes (Control siRNA) were used as controls. The reverse transfection method was adopted for silencing. Briefly, after subculture, BV-2 cells were resuspended in Optimem (GIBCO, Invitrogen, catalogue number 31985070) and plated in 6-well plates at a density of 3 × 10^5^ cells/ml. This was followed by adding 500 μl Optimem with 10 μl siRNA and 4 μl lipofectamine dropwise in the above well. The cells were incubated with the siRNA mix for 8 h and then the medium was replaced with DMEM with 2% FBS without antibiotics and incubated for 40 h and then subjected to hypoxia for 8 h. After that protein was extracted and processed for western blotting. Each group has triple repetition wells and the experiments have been repeated at least in triplicate.

### qPCR

Total RNA was extracted using the RNeasy Mini kit (Qiagen, Valencia, CA, USA; catalogue number: 74104). Reverse transcription reactions were performed using the AMV Reverse Transcriptase system (Promega, Madison, Wisconsin, USA). Primer pairs were designed using Primer 3 software (version 1.0). The mouse gene sequences were as following. TLR4: Forward-ctacctggaatgggaggaca; Reverse-cttagcagccatgtgttcca; β-actin: Forward-ggattccatacccaagaagga; Reverse-gaagagctatgagctgcctga. 2 ml aliquot of each reverse transcription product was added to the 10 ml reaction mixture containing Fast SYBR^®^ Green Master Mix (Invitrogen, Cat. No. 4385612) and 0.5 mM of each primer to amplify the genes in a Fast Real-Time PCR machine (Biosystems 7900HT; Life Technologies biotechnology, Germany). The expression difference of TLR4 between different groups were calculated according to the following formula:

$$\text{-Fold change}=2^{-}{}^{\left[\text{Ct }\left(\text{control}\right)\text{ gene X}-\text{Ct }\left(\text{control}\right)\text{ actin}\right]-\left[\text{Ct }\left(\text{activated}\right)\text{ gene X}-\text{Ct }\left(\text{activated}\right)\text{ actin}\right]\;}.$$

### Western blotting analysis

Culture medium was removed from the culture plate, and cells were washed twice with ice cold PBS. Cells were lysed with lysis buffer, mechanically scraped off with a rubber scraper and centrifuged at 13,000 rpm for 25 mins. Protein concentration was then determined by using a protein assay kit (Bio-Rad, Hercules, CA, USA, catalogue number500–0002). Next, 20 μg of the protein sample was loaded and separated on 10% sodiumdodecyl sulfate-polyacrylamide gels. The proteins embedded in the gel were then transferred to polyvinylidene difluoride membranes using a semidry electrophoretic transfer cell (Bio-Rad). Membranes were washed with TBS-0.1% Tween and then membranes were incubated with 5% nonfat dry skim milk for 30 mins at room temperature. Next, they were incubated with anti-mouse TLR4 (1:1000; Santa Cruz Biotechnology, catalogue number sc-293072), anti-rabbit cleaved caspase3 (1:1000, Cell signaling catalogue number 9661), anti- mouse iNOS (1:1000, BD Pharmingen, San Jose, CA USA, catalogue number 610432) anti-rabbit TNF-α (1:1000, Millipore Bioscience Research Reagents, Billerica, MA, USA, catalogue number AB1837P) and anti-rabbit IL-1β (1:1000, Millipore Bioscience Research Reagents, catalogue number AB1832P), anti-rabbit NF-κB/p65 (1:1000, Santa Cruz Biotechnology, catalogue number sc-109) and anti-mouse β-actin (dilution 1:10,000; Sigma-Aldrich, catalogue number A5441) overnight on a shaker at 4°C. After three washes with TBS-0.1% Tween, the membranes were incubated with horseradish peroxidase-conjugated secondary antibody for 1h. The proteins were detected with a chemiluminescence detection system according to the manufacturer’s instruction (Super signal West Pico Horseradish Peroxidase Detection Kit; Pierce Biotechnology, Rockford, IL, USA, catalogue number 34077). Band intensity was quantified using Image J software. All experiments were repeated at least in triplicate.

### Apoptosis analysis using the Annexin V—FITC conjugation and PI cell staining

FITC Annexin V/Dead Cell Apoptosis Kit with FITC Annexin V and PI for Flow Cytometry kit were used to determine the percentage of cells undergoing apoptosis after hypoxia treatment for 12 h following the manufacturer’s instruction. After staining the cells were analyzed by flow cytometry. The apoptotic cells were defined as showing positive Annexin V staining prior to the appearance of PI staining. Each group has triple repetition wells and the experiments have been repeated at least in triplicate.

### Cell viability analysis of BV-2 cells

The effect of hypoxia on the viability of BV-2 cells was evaluated by CellTiter 96^®^AQueous One Solution Cell Proliferation Assay kit (Promega, Fitchburg, WI, USA, catalogue number G3580). The cell viability of the control cells and those subjected to hypoxia for 12 h was measured by the addition of 3-(4,5-dimethylthiazol-2-yl)-5-(3-carboxymethoxyphenyl)-2-(4-sulfophenyl)-2h-tetrazolium, inner salt (MTS) reagent to each well (20 μl/well) followed by incubation for 4 h at 37°C in a humidified atmosphere of 5% CO_2_ and 95% air. After that the absorbance at 490 nm was measured using a microplate reader (GENIOS, Tecan, Switzerland). Cell viability was expressed as a percentage of control BV-2 cells. Each group has triple repetition wells and the experiments have been repeated at least in triplicate.

### Statistical analyses

The data were presented as mean ± SD. The statistical significance of differences between control, hypoxic and treatment groups was calculated using Student’s *t*-test and one-way analysis of variance (ANOVA). Statistical significance was determined by *p<0.05 and **p<0.01.

## Results

### Caspase3 expression induced by hypoxia controls microglia activation

**Fig 1:**
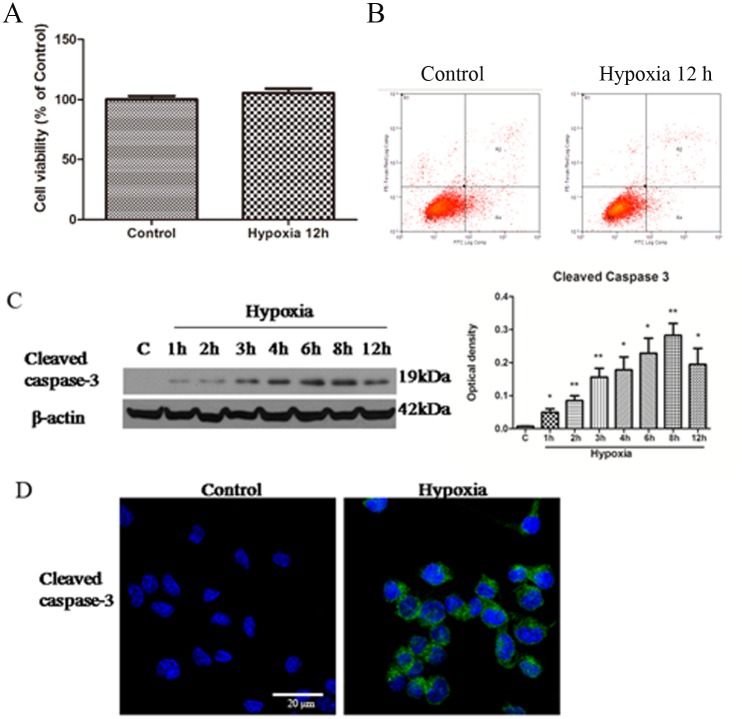
Hypoxia induces caspase3 expression without affecting cell viability in microglia. A. Bar graph shows the viability of BV-2 cells after hypoxia for 12 h compared to control by MTS. Note no significant difference was observed in the two groups. B. PI- Annexin V staining determined by FACS analysis in microglia. C. Western blotting of caspase3 protein expression in BV-2 cells exposed to hypoxia for 2, 4, 6, 8 and 12 h and control (c). The left panel in C shows specific bands of cleaved caspase3 (17 kDa) and β-actin (43 kDa). The right panel in C is a bar graph showing significant changes in the optical density following hypoxic exposure (normalized with β-actin). Note significant increase in cleaved caspase3 expression after hypoxic treatment of varying durations in BV-2 cells, especially at 8 h. D. Confocal images showing cleaved caspase3 expression in control BV-2 cells and those exposed to hypoxia for 8 h. Weak cleaved caspase3 expression is detected in the control BV-2 cells with enhanced immunofluorescence intensity after 8 h of hypoxic exposure. The experiments have been repeated at least in triplicate. Significant differences between control and hypoxic BV-2 cells are calculated using Student’s *t*-test and expressed as *p < 0.05 and **p < 0.*01*. The values represent the mean ± SD in triplicate. Scale bar in D = 20 μm.

BV-2 cells were exposed to hypoxia for 12 h; cell viability was then assessed by MTS. No significant difference in cell viability was found after hypoxia compared with the control in both the MTS assay (Fig 1A) and in the flow cytometry analysis with PI-annexin V double staining kit (Fig 1B). To further investigate the expression of caspase3 in microglia after hypoxia exposure, caspase3 protein expression level in BV-2 cells between the control and hypoxic groups was compared. Caspase3 protein expression level was increased at 4, 6, 8 and 12 h following hypoxic exposure whose expression was most pronounced at 8 h, as revealed by western blot (Fig 1C). Immunofluorescence staining confirmed that caspase3 was weakly expressed in normal BV-2 cells; however, caspase3 immunofluorescence intensity was markedly enhanced after 8 h of hypoxia (Fig 1D).

**Fig 2:**
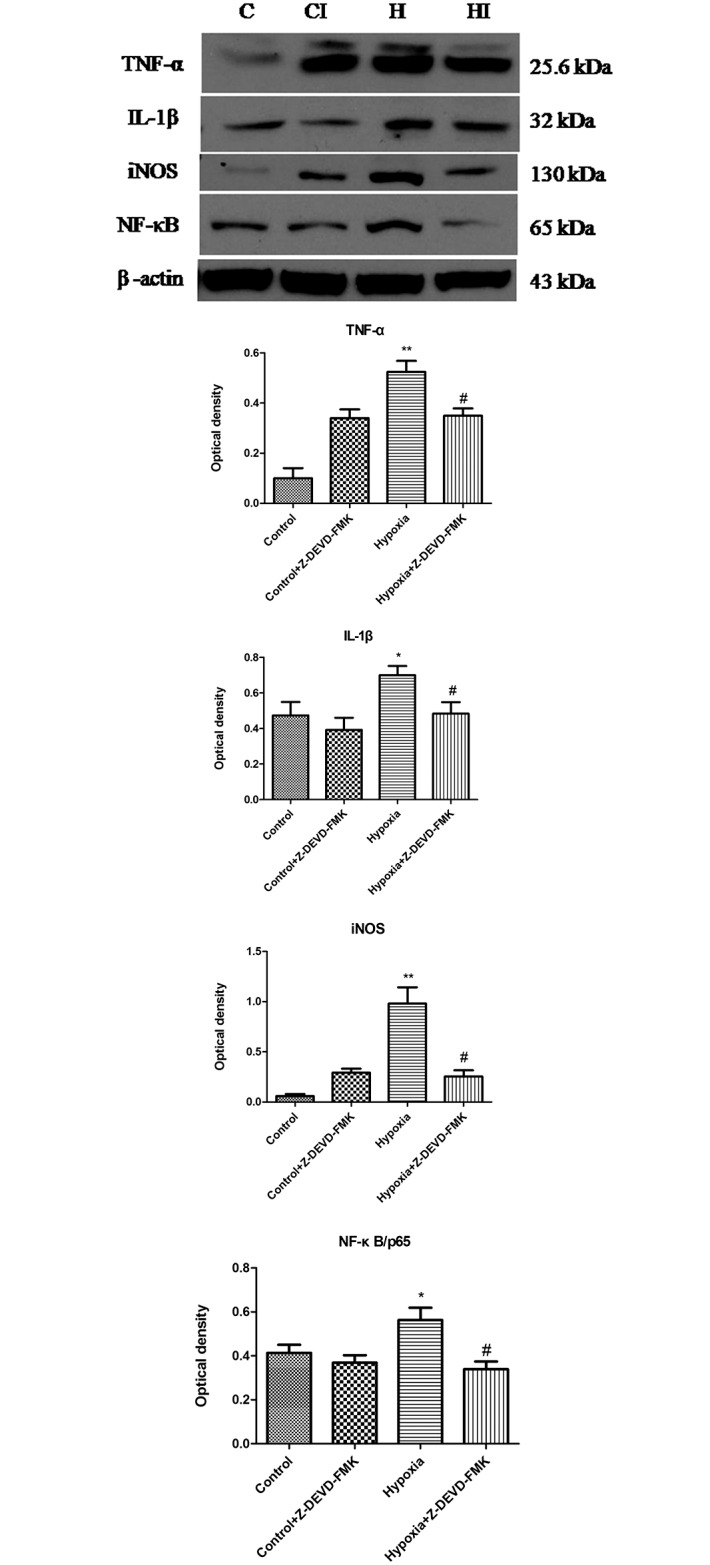
Caspase3 inhibitor reduces release of proinflammatory mediators and NF-κB activation. Western blotting of TNF-α, IL-1β, iNOS and NF-κB protein expression in BV-2 cells following hypoxic exposure and Z-DEVD-FMK pretreatment. The upper panel shows specific bands of TNF-α (25.6 k Da), IL-1β (17k Da), iNOS (130 kDa), NF-κB /P65 (65k Da) and β-actin (43 kDa. The lower panels are bar graphs showing significant changes in the optical density in protein expression of different groups (normalized with β-actin). Note the decrease in TNF-α, IL-1β, iNOS and NF-κB expression in hypoxia+ Z-DEVD-FMK group compared with hypoxic BV-2 cells. The experiments have been repeated at least in triplicate. The statistical significance of differences between different groups was calculated using ANOVA. Significant difference between control vs hypoxia groups is shown as *p<0.05 and **p<0.01; significant difference between control vs hypoxia and hypoxia vs hypoxia+ Z-DEVD-FMK groups is shown as #*p*<0.05 and ##*p*<0.01. The values represent the mean ± SD in triplicate.

Caspase3 activation was reported to mediate microglia activation after stimulation with LPS and lipoteichoic acid. To ascertain the involvement of caspase3 activation in hypoxic microglia, its specific inhibitor Z-DEVD-FMK was applied before hypoxia and the expression of inflammatory factors was then evaluated. It was found that Z-DEVD-FMK significantly repressed the protein expression of inflammatory mediators including TNF-α, IL-1β and iNOS. Furthermore, NF-κB/P65 expression was also inhibited after Z-DEVD-FMK treatment in hypoxic BV-2 cells (Fig 2).

### Caspase3 activation was dependent on TLR4 expression in hypoxic microglia

**Fig 3:**
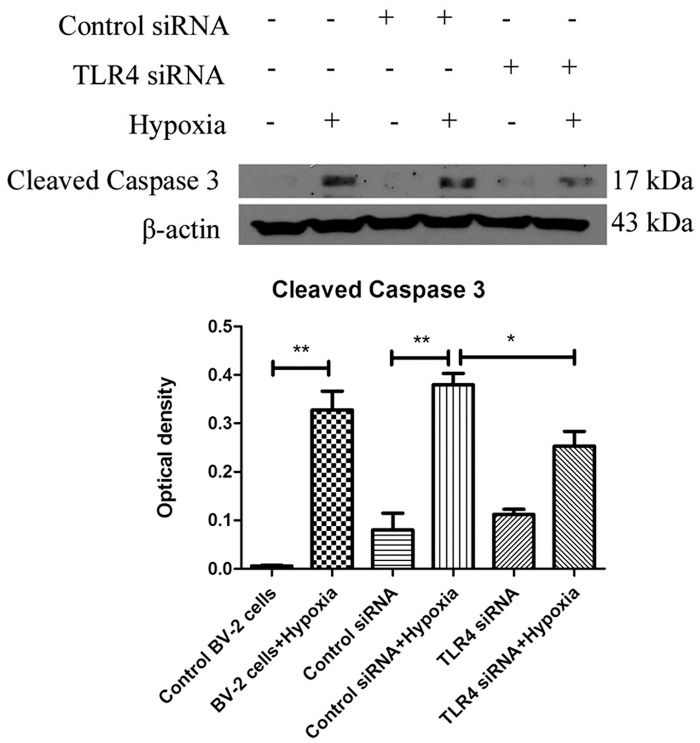
Caspase3 activation is dependent on TLR4 expression in hypoxic microglia. Western blot analysis of caspase3 activation in control BV-2 cells, BV-2 cells + hypoxia, BV-2 cells transfected with control siRNA, BV-2 cells transfected with control siRNA + hypoxia, BV-2 cells transfected with TLR4 siRNA and BV-2 cells transfected with TLR4 siRNA + hypoxia. The upper panel shows specific bands of cleaved caspase3 (17 kDa) in different groups of BV-2 cells given different treatments. The lower panel are bar graphs showing significant changes in the optical density following hypoxic exposure (normalized with β-actin). The experiments have been repeated at least in triplicate. The statistical significance of differences between different groups was calculated using ANOVA. Significant difference between different groups is shown as *p<0.05 and **p<0.01. The values represent the mean ± SD in triplicate.

TLR4 expression was knocked down by specific TLR4 siRNA. TLR4 knockdown efficiency has been confirmed in our previous study [8]. Cleaved caspase3 protein expression was examined by western blot. Hypoxia induced expression of cleaved caspase3 was significantly suppressed in microglia with TLR4 knockdown with siRNA (Fig 3).

### Melatonin inhibited TLR4 and caspase3 activation in hypoxic microglia

**Fig 4:**
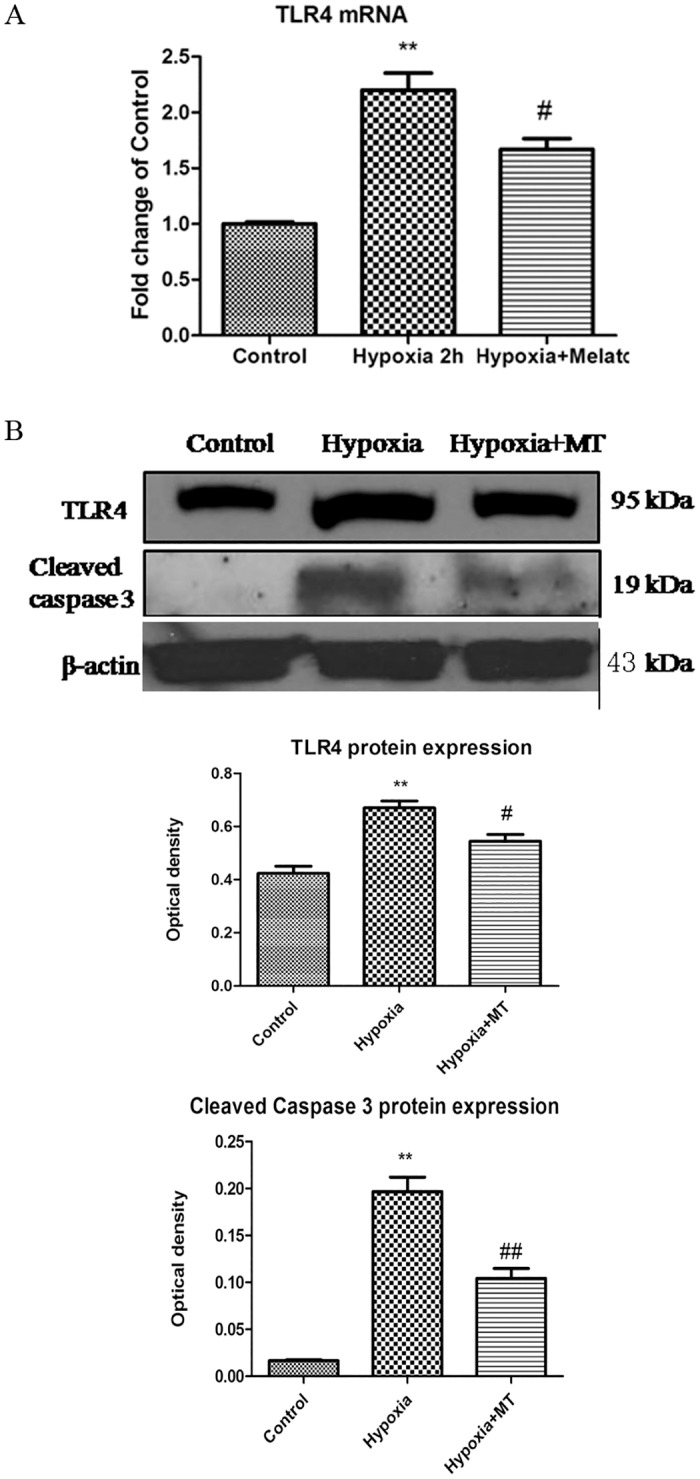
Melatonin inhibits TLR4 mRNA and protein expression and caspase-3 activity in hypoxic BV-2 cells. A. Hypoxia induces TLR4 mRNA expression in hypoxic microglia that is suppressed by melatonin. B. BV-2 cells subjected to hypoxia, followed by western blot analysis of TLR4 and cleaved caspase-3 with or without melatonin treatment. The panel shows specific bands of TLR4 (95 kDa), cleaved caspase-3 (17 kDa) and β-actin (43 kDa). Note TLR4 and cleaved caspase-3 expression in melatonin-treated group is significantly decreased in comparison with the untreated group (normalized with β-actin). The experiments have been repeated at least in triplicate. The statistical significance of differences between different groups was calculated using ANOVA. Significant difference between control vs hypoxia groups is shown as *p<0.05 and **p<0.01; significant difference between hypoxia vs hypoxia +Melatonin groups is shown as #*p*<0.05 and ##*p*<0.01. The values represent the mean ± SD in triplicate.

qPCR analysis showed that TLR4 mRNA expression level was increased by more than two folds in hypoxic BV-2 cells; it was however, decreased by about 20% when treated with melatonin (Fig 4A). TLR4 protein expression pattern followed that of mRNA in melatonin treated BV-2 cells (Fig 4B). Cleaved caspase 3 protein expression was also significantly suppressed by melatonin treatment (Fig 4B).

### Melatonin inhibited TLR4 expression in hypoxic neonatal rat microglia

**Fig 5:**
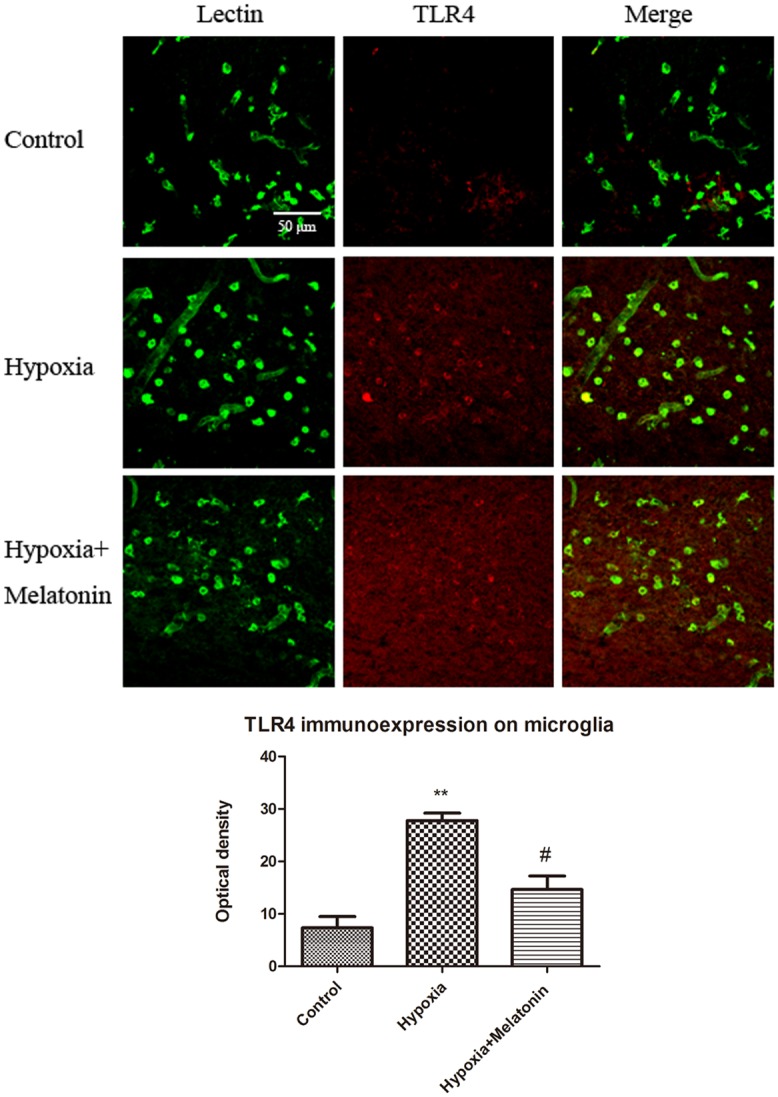
Melatonin suppresses the increase in TLR4 immunofluorescence in microglia in neonatal rats after hypoxic treatment. Confocal images showing TLR4 expression in lectin labeled (green) microglia in the corpus callosum of control, hypoxia and hypoxia+melatonin rats at 3 days after the hypoxic exposure. Increase in TLR4 expression in microglia is evident in hypoxic rats. Note TLR4 immunofluorescence intensity is attenuated in hypoxia+melatonin rats compared with that in the hypoxic rats. The lower graph showing the optical density of TLR4 on microglia of different groups. Note the optical density of TLR4 on microglia was increased in rats following hypoxia challenge and the optical density was decreased in hypoxia+melatonin group compared with hypoxia group. The experiments have been repeated at least in triplicate. The statistical significance of differences between different groups was calculated using ANOVA. Significant difference between control vs hypoxia groups is shown as *p<0.05 and **p<0.01; significant difference between hypoxia vs hypoxia+melatonin groups is shown as #*p*<0.05 and ##*p*<0.01. The values represent the mean ± SD in triplicate. Scale bar = 20 μm.

To investigate the effect of melatonin *in vivo*, melatonin was injected into neonatal rats exposed to hypoxic injury. It is well documented that microglia in the developing brain were amoeboidic in phenotype and were distributed mainly in the corpus callosum above the lateral ventricles. In view of this, we had focused on the microglia in this region in respect to the TLR4 expression. Immunofluorescence labeling showed that TLR4 was weakly expressed in microglia in normal rats. In hypoxic rats, TLR4 immunofluorescence on microglia was noticeably enhanced. However, in hypoxia+Melatonin rats, TLR4 immunofluorescence on microglia was attenuated when compared with that in Hypoxic rats. The optical density analysis of TLR4 on microglia further confirmed the changes (Fig 5).

## Discussion

Neuroinflammation mediated by activated microglia is involved in the pathology of different neurodegenerative diseases and disorders through the expression of cytotoxic mediators [21, 22]. Clarifying the underlying molecular mechanism of hypoxia induced microglia activation is therefore important for modulating microglia activation. Here we found that pro-apoptotic caspase3 was activated in hypoxic microglia in a TLR4 dependent manner. Inhibition of caspase3 pathway effectively blocked microglia activation after hypoxia. Interestingly, pretreatment with melatonin significantly suppressed TLR4 expression and the following caspase3 activation in hypoxic microglia.

Caspases are proteases that are best known for triggering apoptotic cell death [23–25]. However, it has been reported recently a novel, non-apoptotic role for caspases in the activation of microglia and subsequent neurotoxicity [9]. In this connection, it was reported that stimulation of microglia with the pro-inflammatory stimuli LPS, LTA, PamC3sk4 and IFN-c triggers TLR4 dependent-caspase3 activation, surprisingly not leading to cell death. Treatment with an irreversible caspase inhibitor or specific small interfering RNA (siRNA) prevented the activation of microglia. Further experiments have shown that the upstream of caspase3 and caspase7 cleavage was TLR4 dependent [9]. However, as far as can be ascertained, the expression of caspases3 in activated microglia induced by hypoxia has not been explored. The present results have confirmed a similar role of caspase3 in hypoxic microglia with the observation that caspase3 cleavage was increased in activated microglia that was not accompanied by cell death. Arising from the present results, the role of caspase3 in microglia activation induced by hypoxia is further amplified and this would have therapeutic implications. Inhibition of caspase3 with its inhibitor Z-DEVD-FMK significantly decreased the expression of proinflammatory mediators including TNF-α, IL-1β, iNOS and NF-κB. Therefore, inhibition of caspase3 activation specifically may contribute to neuroprotection. We also showed that caspase3 activation was TLR4 dependent. In our previous study [8], we have reported the function of TLR4 in microglia activation after hypoxia. The present study has extended this to illustrate the downstream factors of TLR4, namely, caspase3, in mediating microglia activation.

Recent report has provided evidence that melatonin protects liver against ischemia and reperfusion injury through inhibition of toll like receptor signaling. More importantly, increased levels of TLR3 and TLR4 protein expression induced by ischemia/reperfusion were attenuated by melatonin treatment [20]. We have reported that TLR4 expression mediated neuroinflammation in response to hypoxia in activated microglia [8]. Besides, previous study in our group also showed that melatonin inhibited the release of proinflammatory cytokines in microglia of hypoxic neonatal rats [19]. Arising from this, it is suggested that melatonin acts by a direct suppression of melatonin on TLR4 protein expression followed by that of caspase3 in hypoxic microglia. As a corollary, it is suggested that it would lead to a reduction in release of TNF-α, IL-1β, iNOS and NF-κB.

Another novel finding was the suppression of TLR4 mRNA expression by melatonin suggesting that the neurohormone could suppress transcription of TLR4 in activated microglia. Hypoxia-inducible factor-1α (HIF-1α) was reported to activate TLR4 expression by direct binding to the TLR4 promoter region [26]. It has been documented that melatonin suppressed HIF-1α transcriptional activity thus leading to a decrease in its target gene VEGF expression in HCT116 human colon cancer cell line under hypoxic exposure [27]. However, it is not clear whether melatonin would inhibit the binding of HIF-1α on TLR4 promoter in microglia, and this warrants further investigation.

## Conclusion

This study has provided molecular evidence supporting that melatonin effectively inhibits TLR4 expression and the activation of its novel target caspase3 signaling in hypoxia microglia. The mechanism via which melatonin acts in reducing microglia activation and reduced production of proinflammatory mediators is better clarified. Therefore, modulating the expression of TLR4-dependent caspase3 signaling that governs the expression of proinflammatory mediators may be a potential therapeutic strategy for amelioration of microglia-mediated neuroinflammation that is widely implicated in major neurodegenerative diseases.
